# Analgesic and Anti-Arthritic Activities of Polysaccharides in *Chaenomeles speciosa*

**DOI:** 10.3389/fphar.2022.744915

**Published:** 2022-03-24

**Authors:** Doudou Huang, Shenggui Jiang, Zenan Du, Yanhong Chen, Dan Xue, Xiujuan Wang, Mengshuang Li, Feng Zhang, Wansheng Chen, Lianna Sun

**Affiliations:** ^1^ Department of TCM Processing, Shanghai University of Traditional Chinese Medicine, Shanghai, China; ^2^ Institute of Chinese Materia Medica, Shanghai University of Traditional Chinese Medicine, Shanghai, China; ^3^ School of Pharmacy, Changzheng Hospital, Navy Military Medical University, Shanghai, China

**Keywords:** *Chaenomeles speciosa*, polysaccharides, anti-arthritic, analgesic, MAPK

## Abstract

*Chaenomeles speciosa* (Sweet) Nakai has been long used as a folk medicine for rheumatic diseases treatment. This study aimed to investigate the effects and underlying mechanism of polysaccharides in *Chaenomeles speciosa* (CSP) on the pro-inflammatory cytokines and MAPK pathway in complete Freund’s adjuvant (CFA)-induced arthritis and LPS-induced NR8383 cells. We used acetic acid (HAc)-induced writhing and CFA induced paw edema to determine the analgesic activity and anti-inflammatory activity, respectively. CFA rats were administered CSP (12.5, 25.0, and 50.0 mg/kg) daily for 3 weeks *via* oral gavage. The analgesic test was done using three different doses of the extract (50, 100, and 200 mg/kg). The anti-arthritic evaluation involved testing for paw swelling, swelling inhibition, and histological analysis in CFA rats. Finally, ELISA, western blot, qRT-PCR were done to determine the effect of CSP on the activation of MAPK pathway, production of pro-inflammatory cytokines in lipopolysaccharide (LPS)-stimulated NR838 macrophage cells. In pain models, oral uptake of CSP greatly reduced pain perception. Furthermore, in CFA rats, CSP substantially decreased paw swelling as well as synovial tissue proliferation and inflammatory cell infiltration. In addition, CSP was shown to inhibit pro-inflammatory cytokines (TNF-α, IL-1β, and COX-2) as well as JNK and ERK1/2 phosphorylation in LPS-stimulated NR8383 cells. Thus, pro-inflammatory cytokine secretion and MAPK signaling downregulation promoted the analgesic and anti-arthritic effects of CSP.

## Introduction

Rheumatoid arthritis (RA) is a chronic autoimmune disease, with the pathological features of synovial tissue hyperplasia, pannus formation and cartilage erosion. The typical clinical features of RA are joint swelling, pain, and irreversible joints damage, which eventually lead to severe disability (Bang et al., 2009). Because of the characteristics of high disability rate, poor prognosis, and easy recurrence of RA, it causes heavy economic burden to the patient’s family. Previous study suggested the global incidence of RA is about 0.5–1.0%, with the highest incidence in 30–50 years old (Chung et al., 2016). However, the pathogenesis of RA is still unclear, and the clinical treatment of RA is mainly chemical drugs, such as non-steroidal anti-inflammatory drugs (NSAID), anti-rheumatic drugs (ARD), glucocorticoids, etc (Smolen et al., 2016). Nevertheless, these treatments are expensive, and long-term use of chemical drugs can cause a variety of adverse reactions, such as cardiovascular disease, gastrointestinal bleeding, liver and kidney toxicity, growth inhibition, infection, and tumor risks (Urushibara et al., 2004; Andersson et al., 2008; Grosser, 2017; Chen et al., 2018).

As the number of RA patients increases year by year, people are eager to looking for alternative drugs that are inexpensive, safe and effective, and have fewer adverse reactions to treat RA. Traditional Chinese medicine has received more and more attention because of its remarkable curative effect and few adverse reactions (Newman and Cragg, 2016; Luo et al., 2019). As a class of natural macromolecular substances, polysaccharides have obvious advantages and can exert a wide range of pharmacological effects by participating in the body’s physiological reaction, such as anti-oxidation, hypoglycemic, immune regulation, anti-tumor, anti-inflammatory and other biological activities (Zou and Zhang, 2015).

*Chaenomeles speciosa* (Sweet) Nakai belongs to Rosaceae, mainly distributed in China and Myanmar, and it has been long used as food and medicine in China. Modern pharmacological studies have shown that *C. speciosa* has a variety of biological activities, including immune regulation, anti-inflammatory, anti-tumor, antibacterial and antioxidant effects. As a common medicinal and edible plant, *C. speciosa* is rich in polysaccharides. At present, it has been reported that polysaccharides in *C. speciosa* have antioxidant and anti-inflammatory activities (Li and Chen, 2011; Xie et al., 2015). However, the anti-inflammatory mechanism of *C. speciosa* polysaccharide is not clear.

**FIGURE 1:**
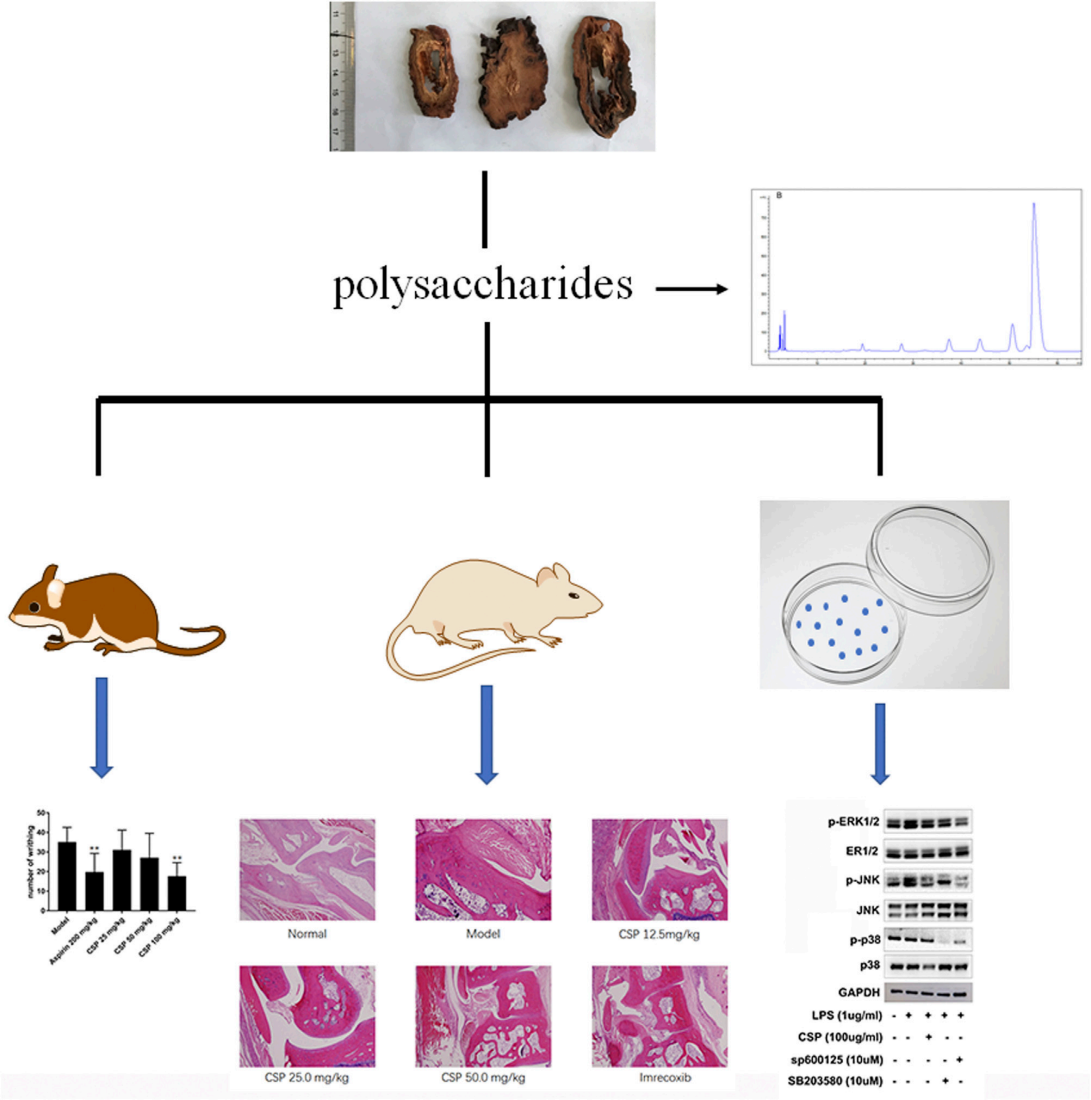
Workflow of analgesic and anti-inflammatory activities of CSP.

Here, we examined the potential medicinal effects of CSP against Complete Freund’s Adjuvant (CFA)-induced inflammation in an arthritic rat model. Additionally, we used LPS-induced NR8383 cells to examine the role of CSP in inflammation. The results showed that CSP exhibited anti-RA activity by suppressing the activation of mitogen-activated protein kinase (MAPK) pathway (Figure 1).

## Materials and Methods

### Preparation of CSP Extract

We collected the fruits of *C. speciosa* from Bozhou, Anhui Province, China, in October 2014 (authenticated by Prof. Hanming Zhang). A voucher specimen (No. CS20141008) for *C. speciosa* fruit was submitted to the authors’ laboratory. We performed hot water extraction of the fruits of *C. speciosa* (100 kg; 1:15), followed by deproteinization using 15% trichloroacetic acid, and precipitation with 70% ethanol. After centrifugation, the precipitate was collected to obtain the crude polysaccharide. The total carbohydrate content of the polysaccharides was determined by the phenol-sulfuric acid method with glucose as standard, and determined the content was 52.97%.

### Monosaccharide Compositions Assays

Mannose (B25303), rhamnose (YY91163), galacturonic acid (B21894), glucose (B21882), galactose (YY91011), and arabinose (B25845) were purchased from Shanghai Yuanye Bio-Technology Co., Ltd. PMP (1-phenyl-3-methyl-5-pyrazolone) pre-column derivatization combined with high performance liquid chromatography (HPLC) were used to determine the monosaccharide compositions of CSP (Cao et al., 2019). The polysaccharide (1.0 mg/ml) was hydrolyzed using 4 M trifluoroacetic acid (TFA) at 110°C for 6 h. The residual TFA was obtained by evaporating the residue under reduced pressure in methanol (2 ml). The hydrolysate and the hybrid standard monosaccharide solution (100 μl, glucose, D-mannose, D-galactose, xylose, maltose, L-arabinose, all 1 mg/ml) were dissolved in 200 μl water; the supernatant was mixed with PMP and 0.3 M NaOH solutions. The reaction was carried out at 70°C for 45 min before being stopped using an HCl solution. The reaction product was removed thrice with chloroform, and the upper layer was centrifuged, and filtered using a 0.22 μM membrane, followed by HPLC analysis. We used the Agilent Eclipse XDB-C18 chromatographic column (30°C), equipped with a UV-detector. The mobile phase was PBS (pH 6.8) mixed with acetonitrile (87: 13) and eluted isocratically for 60 min.

### Animals and Experimental Design

We purchased the Sprague-Dawley (SD) (Male, weight: 120–140 g) rats from the Laboratory Animal Research Center at the Second Military Medical University (SCXK (hu) 2017-0010). The animal study was reviewed and approved by the Animal Ethic Committee of Second Military Medical University (No.CZ201705126) in the accordance with the National Institutes of Health guidelines for animal care. These animals were freely access to food/water and were hosted in rat cages at (24 ± 2)°C, 12 h:12 h light/dark cycle, 40–70% humidity.

Our experimental protocols followed the guidelines of the Animal Guidelines of Second Military Medical University. We followed a previously described method to establish the CFA-induced arthritis model in SD rats (Gupta et al., 2020). First, we measured the volume of the foot (reference), and then intradermally injected CFA (0.1 ml) into the foot pad and recorded as day 0. On day 7, the immunized rats were divided into five group, and then the five groups were administrated with saline (model group), 12.5 mg/kg CSP (low-dose group), 25.0 mg/kg CSP (middle-dose group), 50.0 mg/kg CSP (high-dose group), and 20.0 mg/kg imrecoxib (positive control group) *via* oral gavage, respectively from day 8. Another 10 non-immunized rats were treated with saline (“the control group”). Each group had 10 rats who were treated daily for 3 weeks.

### Paw Edema Measurement

After treatment initiation, foot volume was measured every 3 days and each time the joints were marked based on the initial measurement. The swelling degree and swelling inhibition were calculated based on the swelling volume as follows:

Swelling degree (ΔmL) = paw volume after inflammation—paw volume before inflammation.

Swelling inhibition rate (%) = (paw volume in model group—paw volume in the treatment group)/paw volume in model group × 100%

### Serum Pro-Inflammatory Cytokines Analysis

The pro-inflammatory cytokines in serum, including TNF-α, COX-2, and IL-1β were measured by ELISA kits (Jiancheng Bioengineering, Nanjing, China) according to the manufacturer’s instructions.

### Histopathological Analysis

On day 29, we used isoflurane to sacrifice the rats. The legs below the knee joints (together with the feet) were simultaneously excised, fixed with 10% formalin, and treated with 5% formic acid. The paraffin-embedded tissues were sectioned, stained with H&E stain, and analyzed histopathologically.

### Acetic Acid (Hac)-Induced Writhing

The KM mice (male, weight 23 ± 0.5 g) were purchased from the Laboratory Animal Research Center at the Second Military Medical University and hosted under identical conditions as the SD rats.

The KM mice were divided into five groups (*n* = 10 each): model, aspirin (positive group, 200 mg/kg), low-dose (25 mg/kg CSP), medium-dose (50 mg/kg CSP), and high-dose (100 mg/kg CSP). The animals were administered respective treatments daily for 5 days, and 1 h post-administration, each group was intraperitoneally injected with HAc (10 mg/ml) and observed and recorded for 15 min (Tadiwos et al., 2017).

The following formulas were used to calculate the number of writhing turnovers of the mice in the group, and the writhing inhibition rate:

Writhing Inhibition Rate (%) = (N_1_−N_2_)/N_1_ × 100%

N_1_: Number of writhing in model group; N_2_: Number of writhing in CSP group.

### Cell Culture

We cultured NR8383 murine macrophage cells (Institute of Biochemistry and Cell Biology, Shanghai) in high-glucose DMEM with 10% FBS and 1% antibiotics at 37°C in 5% CO_2_. The CCK-8 assay was used to determine the cytotoxicity of CSP (25.0, 50.0, 100.0, and 200.0 μg/ml) (Yu et al., 2020).

### Determination of mRNA Levels of Cytokines

The Total RNA Kit was used to isolate total RNA from the spleen tissues, followed by quantification via the µ Drop Plate (Thermo scientific, Finland). A Revertaid First Strand cDNA Synthesis Kit was used to synthesize cDNA, followed by qRT-PCR using a SYBR Green Master Mix using corresponding primers for cytokines. The primer sequences were as follows: TNF-α: (F) 5′- GGG​GCC​ACC​ACG​CTC​TTC​TGT​CTA-3′; (R) 5′- CCT​CCG​CTT​GGT​GGT​TTG​CTA​CG -3′), IL-1β (F) 5′- CCT​CTG​TGA​CTC​GTG​GGA​TGA​TG-3′; (R) 5′- CAG​GGA​TTT​TGT​CGT​TGC​TTG​TCT -3′), COX-2 (F) 5′- CTG​GTG​CCG​GGT​CTG​ATG​ATG​TA-3′; (R) 5′- AGC​AGG​TGT​GGG​TCG​AAC​TTG​AG -3′), β-actin (F) 5′- CCT​AAG​GCC​AAC​CGT​GAA​AAG​ATG-3′; (R) 5′- GTC​CCG​GCC​AGC​CAG​GTC​CAG -3′). The following PCR conditions were used: pre-denaturation for 5 min at 95°C, then for 10 s at 95°C, and annealing for 20 s at 60°C for 40 cycles. The data was normalized to β-actin to estimate the relative expression of these genes.

### Measurement of Cytokine Expression

NR8383 cells (1 × 10^5^ cells/well) were cultured in 96-well plates for 24 h, followed by pretreatment with CSP (25, 50, 100, and 200.0 μg/ml) for 12 h, and p38 inhibitor (SB 203580, 10 µM) and JNK inhibitor (SP600125, 10 µM) for 5 h, followed by LPS stimulation (1 μg/ml) for another 30 min. Next, the concentrations of IL-1β, TNF-α, and COX-2 were determined in the supernatant using respective ELISA kits.

### Western Blot

The samples for western blot were prepared following a previously described protocol (Xiong et al., 2019). Briefly, NR8383 cells (1 × 10^5^ cells/well) were cultured in 96-well plates for 24 h, followed by pretreatment with CSP (25, 50, 100, and 200.0 μg/ml) for 12 h, and p38 inhibitor (SB 203580, 10 µM) and JNK inhibitor (SP600125, 10 µM) for 5 h, followed by LPS stimulation (1 μg/ml) for another 30 min. Next, the cells were washed with cold PBS and lysed in ice-cold lysis buffer containing the Mammalian Protein Extraction Reagent. The cell lysates were centrifuged (4°C; 10,000 g; 5 min) and the supernatant was used to measure the expression of MAPKs (p-p38, p38, ERK 1/2, JNK, p-JNK) and GAPDH (Santa Cruz, United States) was used as the internal control. The BCA method was used for protein quantification. After separating the protein specimens on SDS-PAGE, they were transferred to a PVDF membrane, which was treated overnight with GAPDH (1:1,000), ERK1/2 (1:600), p-ERK1/2 (1:400), p38 (1:800), p-p38 (1:600), p-JNK (1:500), JNK (1:500) primary antibodies at 4°C. Next, the membranes were treated with HRP-conjugated secondary antibody. The protein bands were visualized using Pierce^®^ ECL Substrate.

### Statistical Analysis

All experiments were repeated at least thrice and data were represented as means ± SEM. Statistical differences were examined *via* GraphPad Prism 7.0 (GraphPad, La Jolla, CA, United States) by one-way ANOVA, followed by Dunnett’s test.

## Results

### The Monosaccharide Compositions of CSP

**FIGURE 2:**
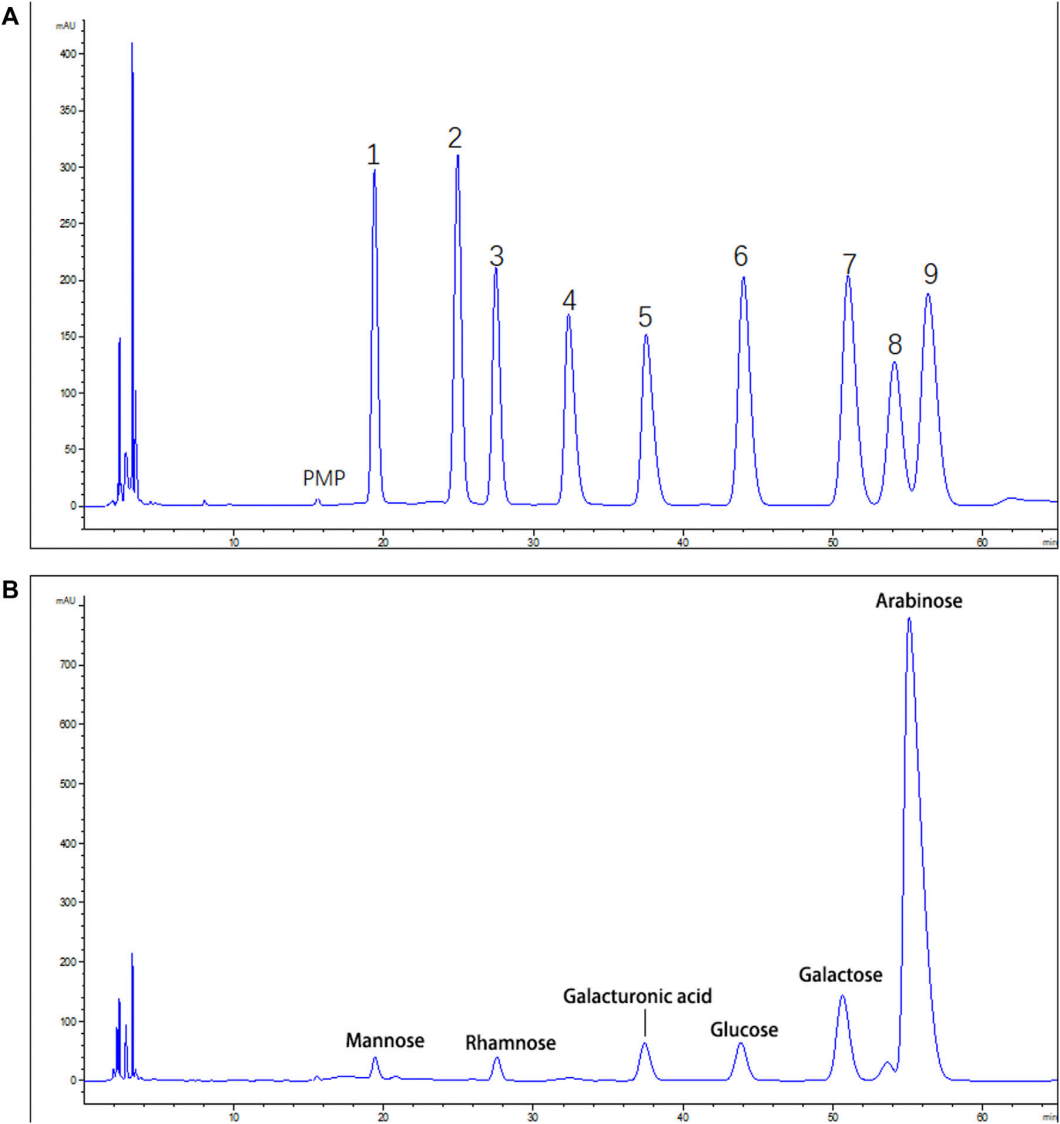
HPLC analysis of CSP **(A)**. Standard monosaccharide mixture. 1: Mannose; 2: ribose; 3: rhamnose; 4: Glucuronic acid; 5: galacturonic acid; 6: glucose; 7: galactose; 8: xylose; 9: arabinose **(B)**. The monosaccharide compositions of CSP.

Monosaccharide content of CSP was evaluated post-PMP derivatization. HPLC chromatogram showed the presence of six peaks (19.12, 27.95, 37.98, 44.01, 51.02, and 56.89 min) (Figure 2B), and were identified as mannose, rhamnose, galacturonic acid, glucose, galactose, and arabinose, according to the references (Figure 2A), which were found to be present in a ratio of 1.66: 2.92: 4.72: 4.25: 9.42: 77.02.

### CSP Attenuates the Development of CFA-Induced Inflammation

**FIGURE 3:**
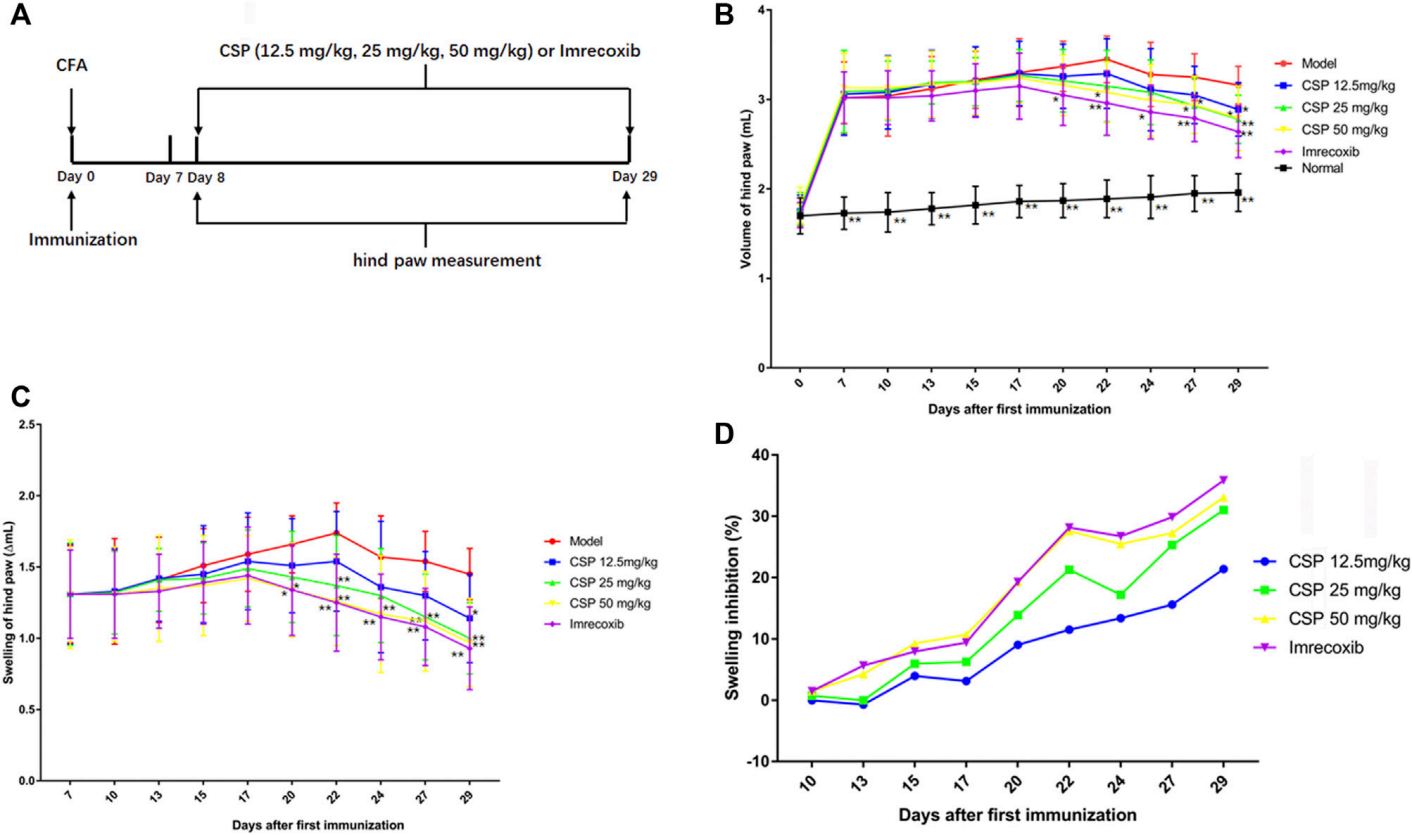
CSP suppressed paw swelling in CFA induced arthritis. The schematic experimental schedule **(A)**, paw swelling **(B, C)**, and swelling inhibition **(D)**. CSP were administered for 3 weeks from the day 8 after the injection of CFA. *p < 0.05, **p < 0.01 compared with model group.

Post-immunization, CFA mice were orally administered CSP (12.5, 25, and 50 mg/kg) from days 8 to 29 to examine the inhibitory effects of CSP (Figure 3A). The swelling volume of the injured paws was used to evaluate CFA-induced disease progression. The rats in the CFA group started showing substantially enhanced paw swelling from day 7 post-injection. Pain swelling was inhibited (*p* < 0.01) after treatment with iremcoxib from day 20 (Figures 3B–C). In the high-dose and medium-dose groups, CSP considerably improved paw swelling from day 22 post-injection of CFA (*p* < 0.01, Figures 3B–C), which improved with time. Additionally, the swelling inhibition rate of the high-dose group was similar to that of the positive control (imrecoxib) from day 22 (Figure 3D).

### Histopathological Analysis

**FIGURE 4:**
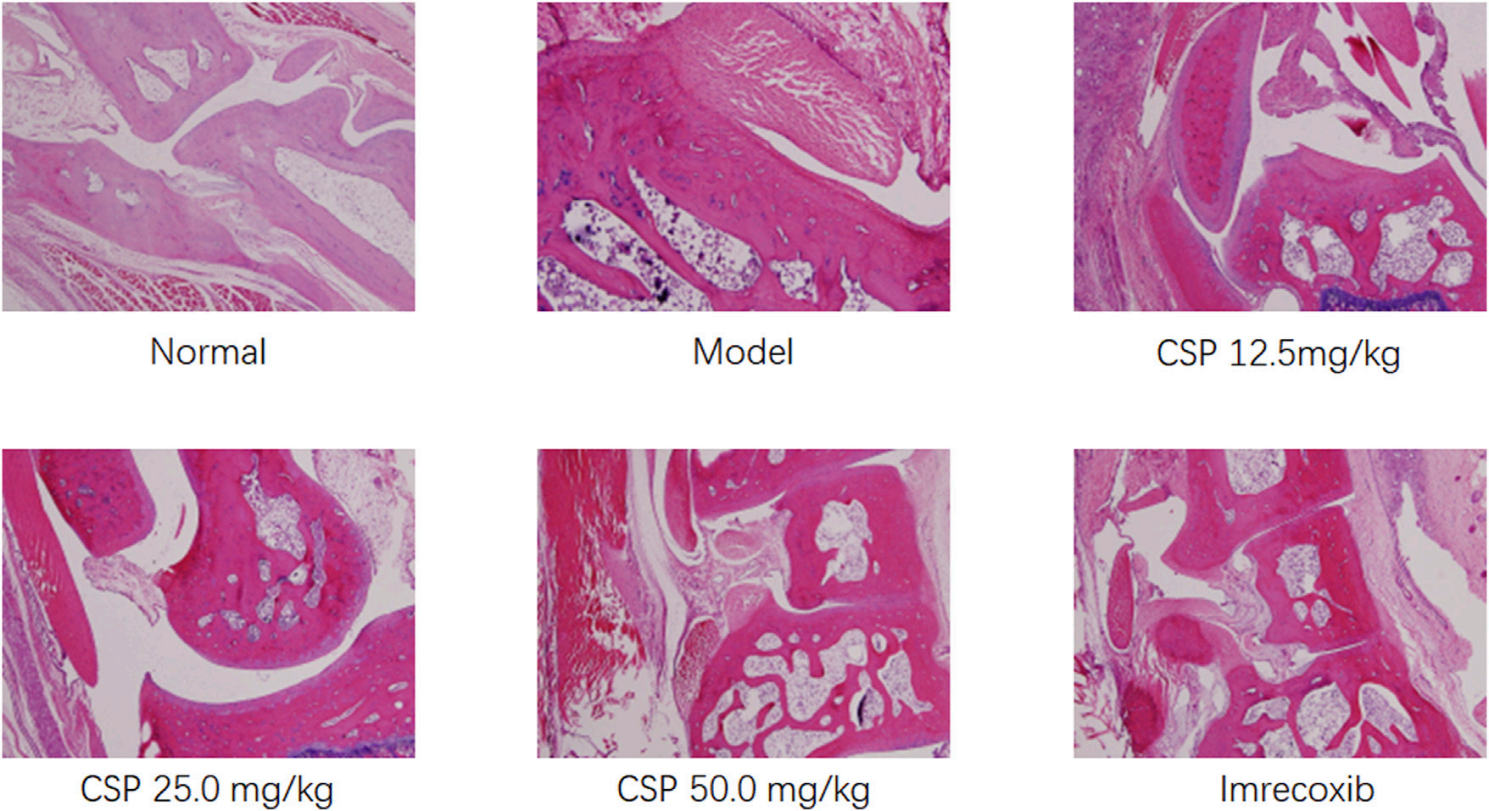
Effects of CSP on the histologic changes in CFA rats (×40).

The results of histopathological examination showed that the model group showed more severe pathological changes compared with normal rats not induced by CFA (Figure4). All rats exhibited various degrees of pathological changes in the ankle joints and feet, mainly due to the proliferation of ankle synovial cells, inflammatory cell infiltration; the articular cartilage and bone tissue in the joint cavity were severely damaged, there was fibrous connective tissue proliferation on the cartilage surface, part of the periosteal fibrosis, and even joint cavity stenosis; obvious soft tissue around the joint inflammation, etc., which suggested the successful establishment of the rheumatoid arthritis model in rats. There was a substantial reduction in the pathological changes in the mouse ankle joint and foot tissue. There was relatively less infiltration of inflammatory cells and proliferation of synovial cells in the ankle joint of rats. The fibrosis of articular cartilage and bone tissue was not severe, and there was no obvious articular surface stenosis. Also, adhesions and inflammation of the surrounding soft tissues were not severe. Additionally, the pathological changes in the 25.0 and 12.5 mg/kg CSP groups were slighter than that in the model group. Thus, CSP could effectively reduce the inflammatory response of the joint synovium and prevent the destruction of cartilage tissue, bone tissue, and tissues around the joint.

Additionally, the degree of pathological changes was quantified (Table 1). The pathological changes in the synovial membrane, articular cartilage, and skin ulcer were divided into four levels, including slight (±), mild (+), moderate (++), and severe (+++). In this study, the number of pathological changes (moderate and severe) in the CSP (50 mg kg−^1^) group were reduced by 33 and 67% in the synovial membrane and skin ulcer, respectively, than the model group. We observed no significant change in the articular cartilage in the CSP (50 mg kg−^1^) group after 3-weeks administration, suggesting that CSP could alleviate pathological changes in CFA-induced rats.

**TABLE 1:** Statistical analysis of abnormal changes of hind paw in different groups.

Group	Number	Pathological changes of synovial membrane				Pathological changes of articular cartilage				Skin ulcer			
		±	+	++	+++	±	+	++	+++	±	+	++	+++
Normal	*n* = 10	0	0	0	0	0	0	0	0	0	0	0	0
Model	*n* = 10	0	7	3	0	3	4	3	0	0	1	4	5
CSP 12.5 mg/kg	*n* = 10	2	4	4	0	1	3	3	0	0	3	5	2
CSP 25 mg/kg	*n* = 10	3	4	3	0	2	5	1	0	2	1	6	1
CSP 50 mg/kg	*n* = 10	2	6	2	0	3	6	0	0	1	6	3	0
Imrecoxib	*n* = 10	2	6	2	0	5	4	0	1	1	6	1	2

Slight (±), Mild (+), Moderate (++), Severe (+++).

### Effect of CSP on Pro-inflammatory Cytokines in Serum

**FIGURE 5:**
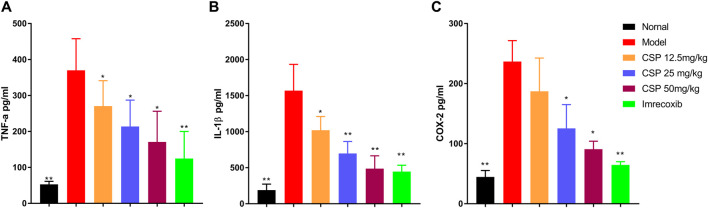
Effects of CSP on pro-inflammatory cytokine expression in the serum of CFA mice. The cytokines IL-1β **(A)**, TNF-α **(B)**, and COX-2 **(C)** were measured by ELISA. The data are presented as the mean ± SEM (*n* = 5). *p < 0.05; **p < 0.01 vs. the CFA model group.

Figure 5 exhibits the production of various pro-inflammatory cytokines in the serum of CFA-induced rats. When compared to the normal group, the model group had higher contents of TNF-α, COX-2, IL-6, and IL-1β in the serum (*p* < 0.01), suggesting that an apparent inflammation existed in the model group (Figures 5A–C). However, the pro-inflammatory cytokines production decreased in the CSP group compared with the model group, especially for the high group with significant difference (*p* < 0.05). These findings suggested that CSP has a significant anti-inflammatory activity in CFA-induced rats.

### Analgesic Activity

**FIGURE 6:**
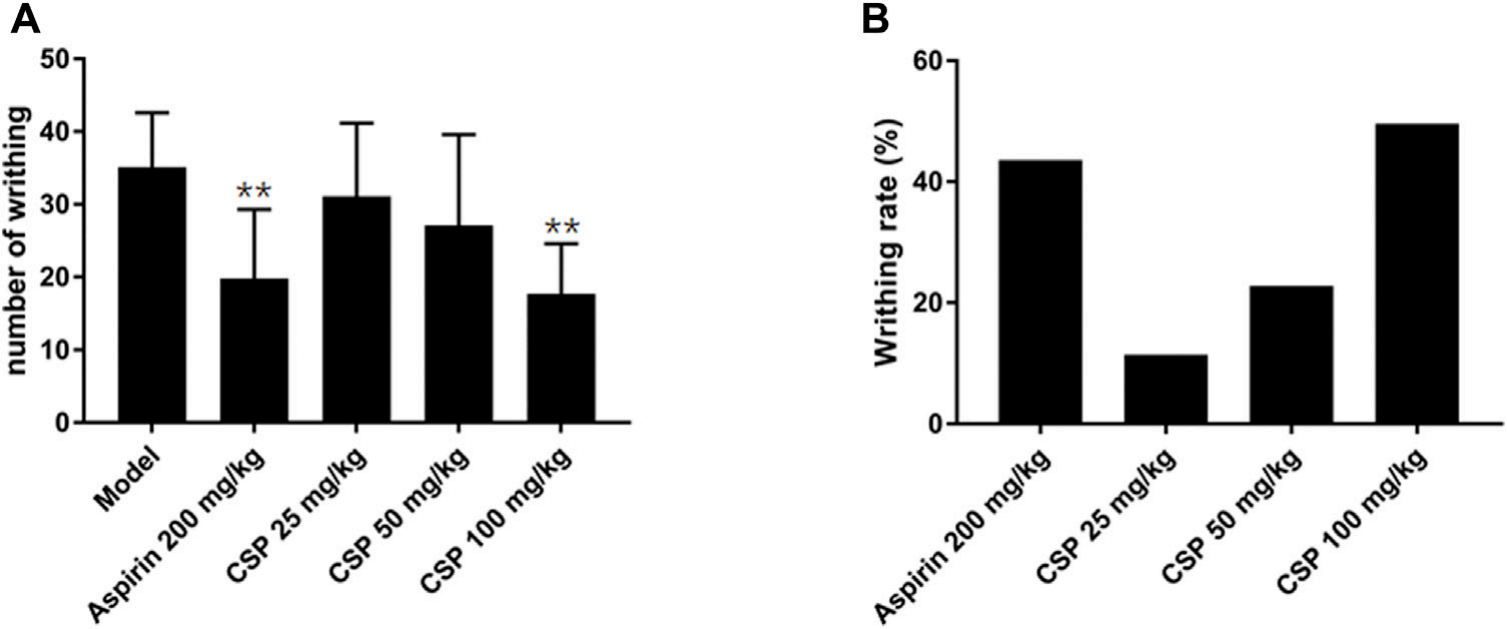
Effect of CSP on acetic acid induced writhing model in mice. **(A)** The number of writhing during 15 min; **(B)** Percentage protection of CSP on acetic acid induced writhing model in mice. **p < 0.01 compared with model group.

The results of HAc-induced writhing method showed that the extract showed substantial analgesic activity. In the HAc-induced writhing method, we found that CSP dose-dependently promoted a reduction in writhing movement in mice compared with the model group; especially for the high-dose group (100 mg/kg), which showed a significant difference (*p* < 0.01) (Figure 6A). In addition, 200 mg/kg Aspirin showed significant difference in reduction in writhing movement (*p* < 0.01), compared with the model group. Moreover, the writhing inhibition rate of CSP (100 mg/kg) was around 43.2%, which is higher than the positive control group (Aspirin 200 mg/kg).

## Effect of CSP on the Production of Proinflammatory Cytokines and Their mRNA Levels NR8383 cells.

**FIGURE 7:**
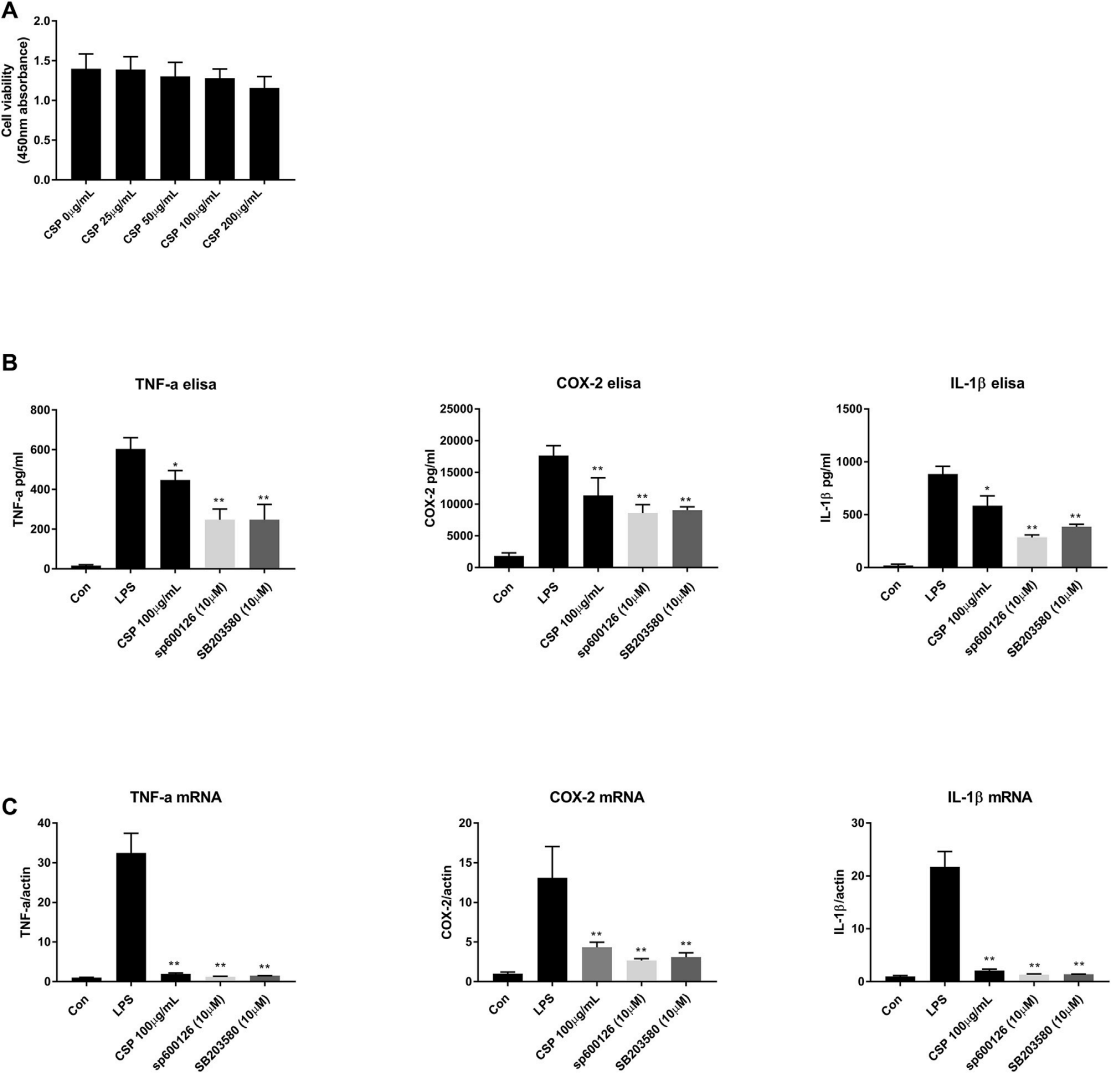
CSP inhibited the pro-inflammatory cytokines in LPS-stimulated NR8383 cells. **(A)** Safe concentration test for CSP in NR8383 cells; **(B)** mRNA expression of IL-1β, TNF-α, and COX-2 in LPS-stimulated NR8383 cells; **(C)** Expression of IL-1β, TNF-α, and COX-2 in LPS-stimulated NR8383 cells. *p < 0.05, **p < 0.01 compared with LPS group.

Here, we studied the effect of CSP on the production of proinflammatory cytokines *in vitro*. Firstly, CCK-8 was used to screen the safe dosage of CSP (0–200 μg/ml), and there was no obvious cytotoxicity (Figure7A). Thus, we chose 100 μg/ml for followed experiment. Then, qRT-PCR was done to estimate the mRNA expression of a few related cytokines in NR8383 cells after incubation with 100 μg/ml CSP. The results demonstrated a substantial increase in the mRNA expression of IL-1β, TNF-α, and COX-2 in the LPS group (*p <* 0.01) than the normal group (Figure 7B). Additionally, CSP could downregulate the mRNA levels of IL-1β, TNF-α, and COX-2 (*p <* 0.01), which were similar to sp600126 and SB3580. After that, ELISA was used to evaluate the contents of IL-1β, TNF-α, and COX-2 in the supernatant of NR8383, which were decreased after CSP treatment (Figure 7C).

### Effect of CSP on the MAPK Signaling Pathway

**FIGURE 8:**
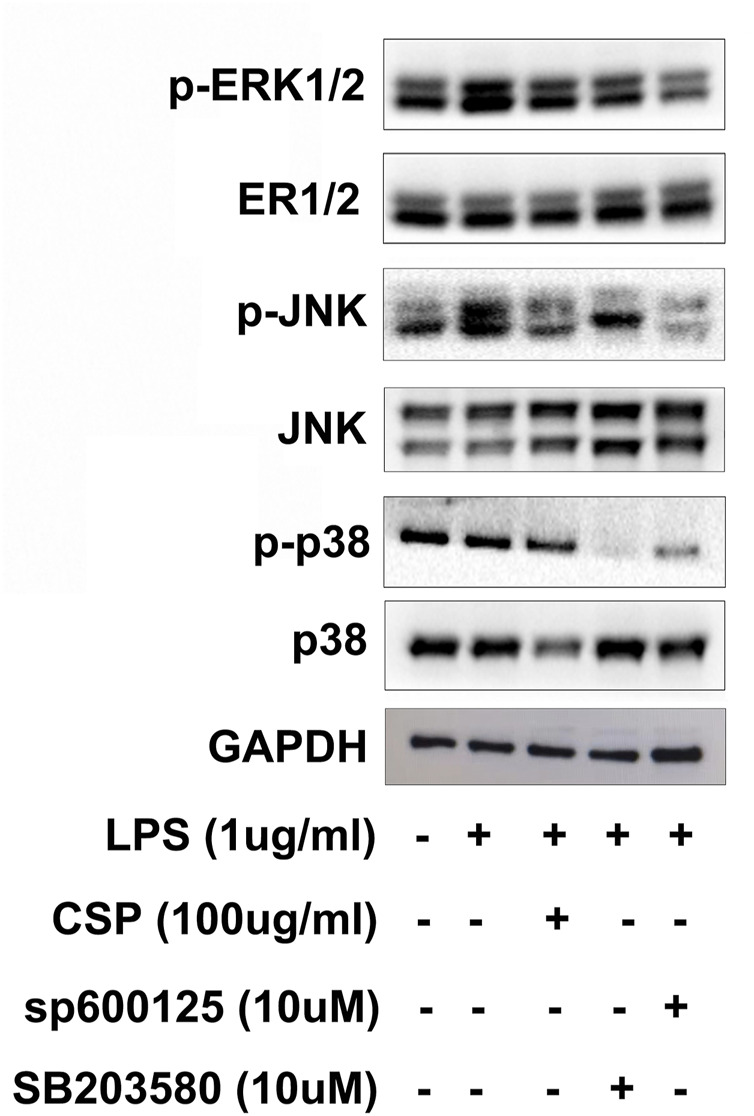
CSP inhibited the phosphorylation of MAPKs in LPS-stimulated NR8383 cells. NR8383 cells were pretreatment with CSP (100 μg/ml) for 12 h, and p38 inhibitor (SB 203580, 10 µM) and JNK inhibitor (SP600125, 10 µM) for 5 h, followed by LPS stimulation (1 μg/ml) for another 30 min. GAPDH was used as loading control.

We performed western blot to test evaluate the potential mechanism involved in CSP-induced attenuation of arthritis. In order to explain whether the improvement of inflammation by CSP is related to MAPK signaling pathway, the key proteins including extracellular signal regulated kinase (ERK), p-ERK, p38 mitogen activated protein kinase (p38), p-p38, c-Jun N-terminal kinase (JNK) and p-JNK were measured (Figure 8). The results of this study demonstrated that the protein expression levels of p-ERK, p-JNK were significantly elevated in the model group. Furthermore, CSP degraded the protein expression levels of p-ERK and p-JNK, while p-p38 showed no significance after CSP treatment. Moreover, when NR8383 cells were pretreated with SP600125, a specific JNK inhibitor, this increase was markedly inhibited, which was similar to the effects of CSP treatment. Similar results were obtained when the inhibitor was replaced by SB203580, which is a broad-spectrum inhibitor of p38 (Figure 8).

## Discussion

*C. speciosa* is a TCM, which was first recorded in the book “Ming Yi Bie Lu” (名医别录). It is commonly used in the treatment of severe waist and knee joint pain. “Ben Cao Shu Jing” (本草经疏) also recorded that *C. speciosa* could dredge meridian and remove dampness. Polysaccharides, one of the main chemical components found in *C. speciosa*, are known to have various pharmacological activities, including anti-tumor potential, immunological, and anti-diabetic effect. In this study, we used CFA-induced arthritis in rats and writhing experiments in mice to investigate the medicinal effects of CSP; the LPS-induced NR8383 cell model was used to explore the effects of CSP on the MAPK signaling pathway.

Inflammation is a normal defensive response that involves the release of pro-inflammatory cytokines. Long-term and chronic inflammation, on the other hand, might be detrimental, causing fever, asthma, atherosclerosis, joint diseases, neurodegeneration, and even cancer (Palladino et al., 2003; Pu et al., 2014). Currently, anti-inflammatory medications, both steroidal and non-steroidal, are used to treat inflammation. However, steroidal anti-inflammatory drugs have the potential to cause elevated blood pressure, osteoporosis, immunosuppression, Cushing’s syndrome, etc., while non-steroidal anti-inflammatory drugs may cause hemorrhagic gastritis, liver toxicity, asthma, and other side effects. These side effects question the long-term use of steroidal and non-steroidal anti-inflammatory medications (Ma et al., 2016). Natural products are secondary metabolites synthesized in different species with distinct chemical structures, indicating their significance as candidates for drug molecules. The natural source-derived polysaccharides are composed of >10 monosaccharide molecules, with different connectivity and a complex molecular structure. Recently, polysaccharides have been used as safe, highly efficient, anti-inflammatory, immune regulators, etc. (Guo et al., 2018).

The pain sensation, as another complication of inflammation, is always accompanied by tissue disruption, and the degree of pain depends on the type of trauma, the healing process as well as the other immune factors. Analgesics are a type of drugs used for relieving pain; thus, they might be useful in the process of short-term wound healing (McHugh and McHugh, 2000; Ronchetti et al., 2017). Pain treatment includes the evaluation of the cytokines and other immune molecules involved in the pain mechanism, which cause inflammation, mediating the formation of wounds and local pain (Widgerow and Kalaria, 2012; Aquino et al., 2013). The analgesic activity of CSP was determined using HAc-induced writhing methods. However, the HAc-induced writhing method, is commonly used to assess peripheral analgesic activity (25). During constriction, the tissue phospholipids release arachidonic acid (AA) *via* COX pathway (Muhammad et al., 2012; Jing et al., 2019). The nociceptive effect of HAc could be prevented using NSAIDs. Aspirin, a COX inhibitor, eliminates inflammatory mediators in the peripheral tissues, thus inhibiting HAc-induced pain. Here, high-dose CSP (100 mg kg−^1^) substantially decreased the writhing in mice in response to intraperitoneal administration of HAc, with a greater effect compared with aspirin (15). The analgesic effect of CSP was probably mediated *via* the inhibition of local peritoneal receptors or COX-related AA pathways.

The CFA-induced rat foot swelling model has been commonly used for the identification of therapeutic drugs of RA in the past (Paunovic and Harnett, 2013; Saleem et al., 2020). After injecting CFA, a gradual increase in the pathological manifestations of RA was observed. Here, CSP substantially improved these pathological changes and paw swelling, indicating that CSP exhibited anti-RA effect. The elevated levels of proinflammatory cytokines, such as IL-1β, TNF-α, and COX-2, could aggravate the progression of RA and could contribute to synovial inflammation and cartilage damage (29). Therefore, inhibition IL-1β, TNF-α, and COX-2 expression could be a target for RA treatment. Here, CSP declined IL-1β, TNF-α, and COX-2 levels, indicating that the anti-RA effect of CSP was directly responsible for the inhibition of secretion of proinflammatory cytokines.

**FIGURE 9:**
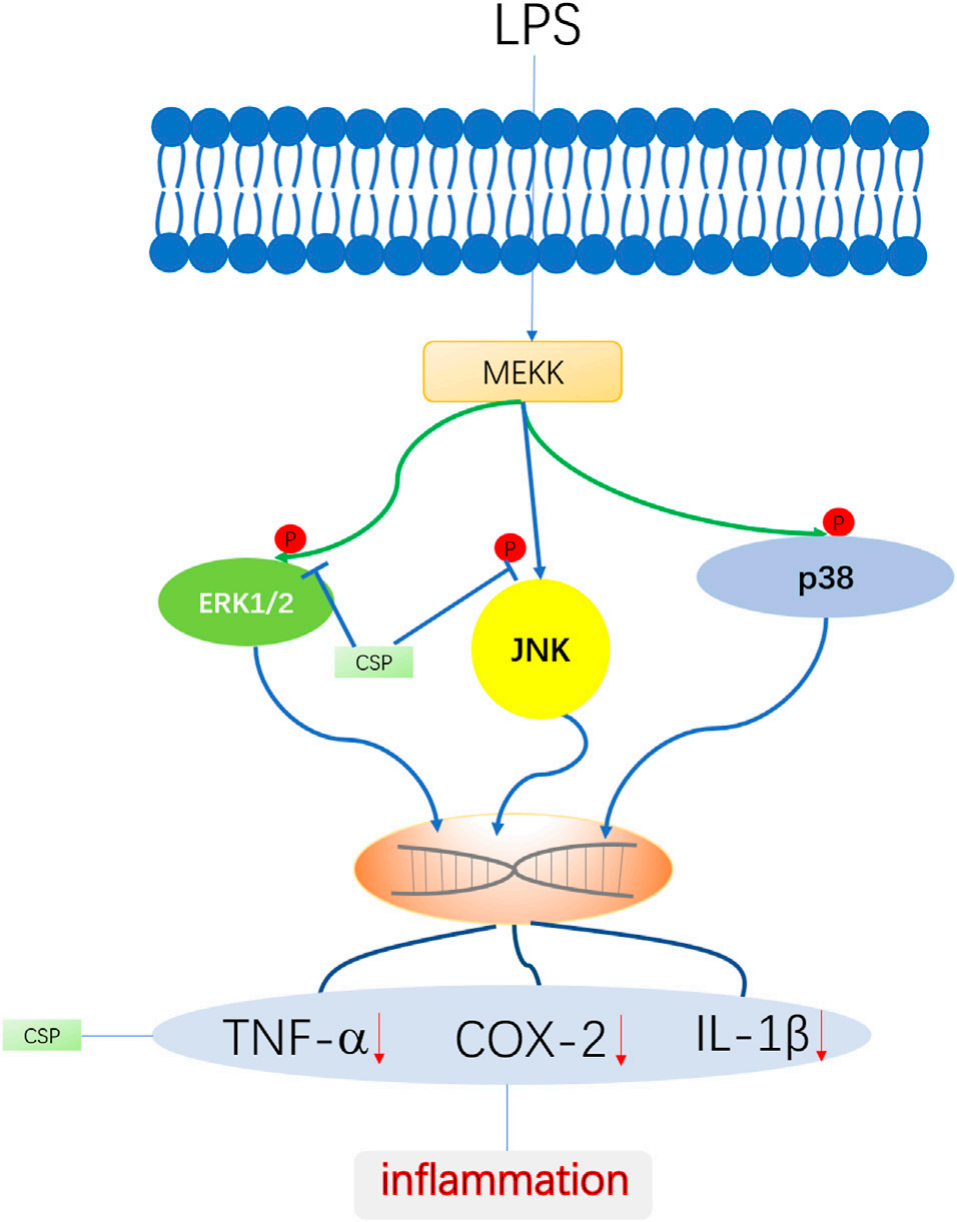
Schematic diagram of anti-inflammatory role of CSP in LPS-induced NR8383 cells. CSP ameliorates inflammation *via* regulating of ERK and JNK phosphorylation.

MAPK is a family of serine/threonine protein kinases involved in the inflammatory process, including ERK, JNKs, and p38 (Wu et al., 2016). Wu et al. discovered that a polysaccharide found in *Sargassum cristaefolium* inhibited iNOS expression by inhibiting p38, ERK, and JNK phosphorylation in LPS-stimulated RAW264.7 cells (Meier and McInnes, 2014). The MAPK signaling pathway molecules ERK and p38 are involved in the pathogenesis of RA (Liang et al., 2012). Our results indicated that CSP could inhibit MAPK signaling pathway activation by preventing the phosphorylation of JNK, and ERK (Figure 9). Previous studies have suggested that MAPK activation was essential for COX-2 expression in peripheral inflammation (Xu et al., 2010), thus, the MAPK signal transduction pathway is partly involved in the regulatory mechanism of this analgesic effect. In our research, we both examined the COX-2 level in CFA-induced arthritis and LPS-induced NR8383 cells, which were both decreased by CSP. Moreover, the phosphorylation of JNK and ERK1/2 were inhibited by CSP, which suggested the analgesia effect of CSP might evolved in regulation of MAPK pathway. However, the role of each MAPK component in effects of CSP on NR8383 cell remains unclear, and an activator of MAPK signaling pathway to antagonize the effects of CSP is not designed in our study. So, further investigations on the specific molecular mechanism of CSP are encouraged.

## Conclusion

Thus, our results showed that MAPK inhibition mediated the beneficial effects of CSP on hind paw swelling and HAc-induced writhing. The anti-inflammatory effect of CSP showed its potential application for treating rheumatoid arthritis.

## Data Availability

The original contributions presented in the study are included in the article/supplementary material, further inquiries can be directed to the corresponding authors.
